# Integrative transcriptomic and metabolomic analysis reveals alterations in energy metabolism and mitochondrial functionality in broiler chickens with wooden breast

**DOI:** 10.1038/s41598-023-31429-7

**Published:** 2023-03-23

**Authors:** Ziqing Wang, Erin Brannick, Behnam Abasht

**Affiliations:** Department of Animal and Food Sciences, University of Delaware, Newark, Delaware, USA

**Keywords:** Agricultural genetics, Metabolic disorders

## Abstract

This integrative study of transcriptomics and metabolomics aimed to improve our understanding of Wooden Breast myopathy (WB). Breast muscle samples from 8 WB affected and 8 unaffected male broiler chickens of 47 days of age were harvested for metabolite profiling. Among these 16 samples, 5 affected and 6 unaffected also underwent gene expression profiling. The Joint Pathway Analysis was applied on 119 metabolites and 3444 genes exhibiting differential abundance or expression between WB affected and unaffected chickens. Mitochondrial dysfunctions in WB was suggested by higher levels of monoacylglycerols and down-regulated genes involved in lipid production, fatty acid beta oxidation, and oxidative phosphorylation. Lower levels of carnosine and anserine, along with down-regulated carnosine synthase 1 suggested decreased carnosine synthesis and hence impaired antioxidant capacity in WB. Additionally, Weighted Gene Co-expression Network Analysis results indicated that abundance of inosine monophosphate, significantly lower in WB muscle, was correlated with mRNA expression levels of numerous genes related to focal adhesion, extracellular matrix and intercellular signaling, implying its function in connecting and possibly regulating multiple key biological pathways. Overall, this study showed not only the consistency between transcript and metabolite profiles, but also the potential in gaining further insights from analyzing multi-omics data.

## Introduction

Concurrent with the rapid growth and high muscle yield, modern broilers are prone to muscle diseases that can negatively affect the well-being of the birds as well as meat quality, such as Wooden Breast myopathy (WB)^1,2^. WB is characterized by pale focally or diffused stiffened areas from cranial to caudal regions on the pectoralis major (p. major) muscle with or without petechial hemorrhage^2–4^. WB is mostly subclinical and asymptomatic, but it can greatly deteriorate muscle health and meat quality, causing a significant negative economic impact to the poultry industry^2,5^.

Although the etiology of this disease is still under investigation, previous research has established association between certain factors and WB. Two main conditions are localized hypoxia and oxidative stress, as suggested by RNA-sequencing and metabolomic studies^6–8^. Oxidative stress is initiated by excess of reactive oxygen species (ROS), which induce long-term cellular damage and impair muscle contractility^6^. The high growth rate of commercial broilers is achieved by allocating more energy towards anabolic growth rather than other key metabolic processes, which could eventually have a negative effect on energy-demanding activities such as muscle contraction^9^. Specifically, altered energy homeostasis and partitioning in broilers may exhibit as dysregulated metabolism of energy substrates including lipid and glucose^7^, resulting in mitochondrial damage and ROS buildup. Various studies have observed susceptibility to WB in fast-growing chickens^7,8,10^, suggesting the importance of energy metabolism to the disease development.

Incorporation of different levels of biological variation has great potential in improving our understanding of mechanisms behind complex traits and diseases. Banerjee et al. applied integrated metabolomic and RNA-seq analysis to identify novel gene-metabolite pairs related to feed efficiency in pigs^11^. A similar approach has also been applied to identify biomarkers for type II diabetes, as well as gaining insights into pathophysiology of metabolic diseases^12^.

This study aimed to advance current understanding of biological mechanisms behind the WB pathology with the long-term goal of mitigating the disease. Previously, WB has been studied using one omics-technique at a time^7–9,13–15^. However, combining both transcriptomics and metabolomics allows us to link gene expression to its metabolic products, showing a bigger picture by studying multiple layers of information. Our integrated metabolomic and RNA-seq analyses revealed that WB is associated with alterations in amino acid and energy metabolism, as well as mitochondrial functionality. These changes in metabolism combined with impaired antioxidant capacity have possibly contributed to adverse myofibrillar changes and oxidative stress in WB. Additionally, our weighted gene-co-expression network analysis (WGCNA) identified the focal adhesion pathway as a strong driving factor for WB status distinction. To the best of our knowledge, there has been no published work on WB combining these two omics data.

## Methods and materials

### Chicken experiment and tissue collection

This research used publicly available data from our laboratory^6,7^, and no new animal experiment was conducted for the current study. However, for the benefit of readers, we provided a description of the animal experiment. Chickens in this study were all males and sampled real time during necropsy of about 300 birds from a commercial broiler line (referred to as Line 2 in the previous study from our laboratory^7^) with high breast muscle yield at Heritage Breeders (Princess Anne, MD), where birds were fed ad libitum and housed under optimal industry growing standards. At 47 days of age, the p. major muscle of live chickens was clinically examined by manual palpation. Specifically, chickens exhibiting severe or moderate p. major muscle firmness were classified as affected with WB, whereas those without any palpable signs of firmness were classified as unaffected. Birds were euthanized by cervical dislocation, and the p. major muscle from each bird was visually observed for macroscopic gross lesions such as areas of hemorrhage, firm and discolored muscle tissue at necropsy to confirm the WB affected or unaffected classification. After gross examination, roughly 1–2 g of muscle tissue were harvested from the caudal aspect of the right p. major muscle from 8 WB affected and 8 unaffected birds with comparable breast muscle weight, immediately frozen in liquid nitrogen, and stored at − 80 °C until further processing. For tissue processing, the frozen breast muscle samples were pulverized within plastic bags by hammering. Two sub-samples from the same pulverized tissue were taken, one for RNA-seq and the other for metabolomic analysis. Tissues were maintained in the frozen state during handling and processing. All methods were carried out in accordance with relevant guidelines and regulations. The animal protocol (#44 12-15-13R) for this experiment was approved by the University of Delaware Agricultural Animal Care and Use Committee.

### RNA-seq samples

Out of 16 total samples, 5 affected and 6 unaffected ones were processed for RNA sequencing as described previously by Mutryn et al.^6^. Obtained RNA-seq data are available at the Sequence Read Archive via accession number NCBI-SRA: SRP224368. Downstream analysis of RNA-seq data for this study was performed in the Biomix High Performance Computing Cluster^16^ at the Delaware Biotechnology Institute, University of Delaware. Raw sequence reads underwent quality check using FastQC v0.11.9^17^ and were mapped to the current chicken reference genome Gallus_gallus-6a (Ensembl, database version 99) using Hisat2 v2.2.0^18^, followed by HTSeq v0.11.2^19^ to categorize the mapped reads using default parameters.

### Metabolomics analysis

Since variance in metabolomics data was expected to be larger than that in RNA-seq data, all 16 breast muscle samples, 8 affected and 8 unaffected, were used for metabolomics profiling by Metabolon Inc. (Durham, NC), as described in prior publication from our laboratory^7^. Briefly, Metabolon utilized liquid chromatography mass spectrometry and a large, well-annotated spectral library to identify the metabolites^20^. Metabolites with missing value across more than 80% of samples were excluded for future analysis since missing values in metabolite data are often not random (Figure S1) but fell below the detection limit threshold^21^. Subsequently, the data were log2 transformed, standardized, and missing values were replaced by the minimum value observed for each metabolite. These data processing steps were performed in R v3.5.2^22^. Numeric representations of the spectral entries for the metabolites identified in our sample set are provided as a supplementary file in Abasht et al.^7^.

### Sample consistency

Sample consistency was assessed by the Principal Component Analysis (PCA) without centering or scaling the variables using R stats package (v3.5.2)^22^. PCA analysis placed the sample C51 which had been clinically and grossly classified as “unaffected” adjacent to the affected samples (Figure S2), suggesting a pattern consistent with WB samples based on both metabolomic and transcriptomic profiles even though this sample exhibited no gross lesions or palpable stiffness. This is consistent with the previous studies where this sample was denoted as a subclinical WB case^6,7^. For the purpose of improving statistical power and accuracy, status of sample C51 was moved from the unaffected to the affected group for further analysis.

### Statistical analysis

HTSeq count data were filtered for low count genes based on the number of chickens in each group, followed by normalization and differential expression analysis using edgeR v3.24.3^23^. The aforementioned analysis and one-way analysis of variance (ANOVA) of metabolites were performed in R v3.5.2^22^. Differentially expressed genes (DEGs) and differentially abundant metabolites were then submitted to MetaboAnalyst v5.0^24^ for analysis using the Joint Pathway Analysis module, where integrated metabolic pathways consisting of both metabolites and metabolic genes were used as a database. Both tight and loose integration methods were performed in order to be comprehensive. Specifically, genes and metabolites were pooled together in a single query for the tight integration method, whereas for the loose integration method, separate analyses were performed for each list and individual p values were weighted at the pathway level before combining to account for different sizes of input data^24^.

To exploit novel relationships between genes and metabolites pertinent to WB, weighted gene co-expression network analysis (WGCNA)^25^ was also performed. This time neither transcriptomic nor metabolomic data were filtered for differential metabolite abundance or differential gene expression. For data processing, transcriptomic data underwent filtering low count genes and by interquartile range, while metabolomic data only underwent missing value filtering and imputation by minimum value. Then, the two datasets were merged at the sample level followed by variance stabilization through log2(x + 1) transformation and standardization. WGCNA was performed in R v3.5.2^22^ using package WGCNA v1.70-3^26^. A soft threshold of 9 was selected based on the recommendation for small sample size from developers^27^. To consider both positive and negative correlations between features, unsigned networks were constructed with a minimum module size of 20 and module eigengene dissimilarity threshold of 0.15. To visualize the relationships among modules and WB, an eigengene network was constructed in R v3.5.2^22^ using package WGCNA v1.70-3^26^. Candidate modules were selected based on the module size, significant correlation (FDR < 0.05) with the WB status and inclusion of both genes and metabolites. Hub features in a module were identified as those with the highest module membership (MM) and gene significance (GS) values. The study was carried out in compliance with the ARRIVE guidelines.

## Results

### Joint pathway analysis

**Figure 1:**
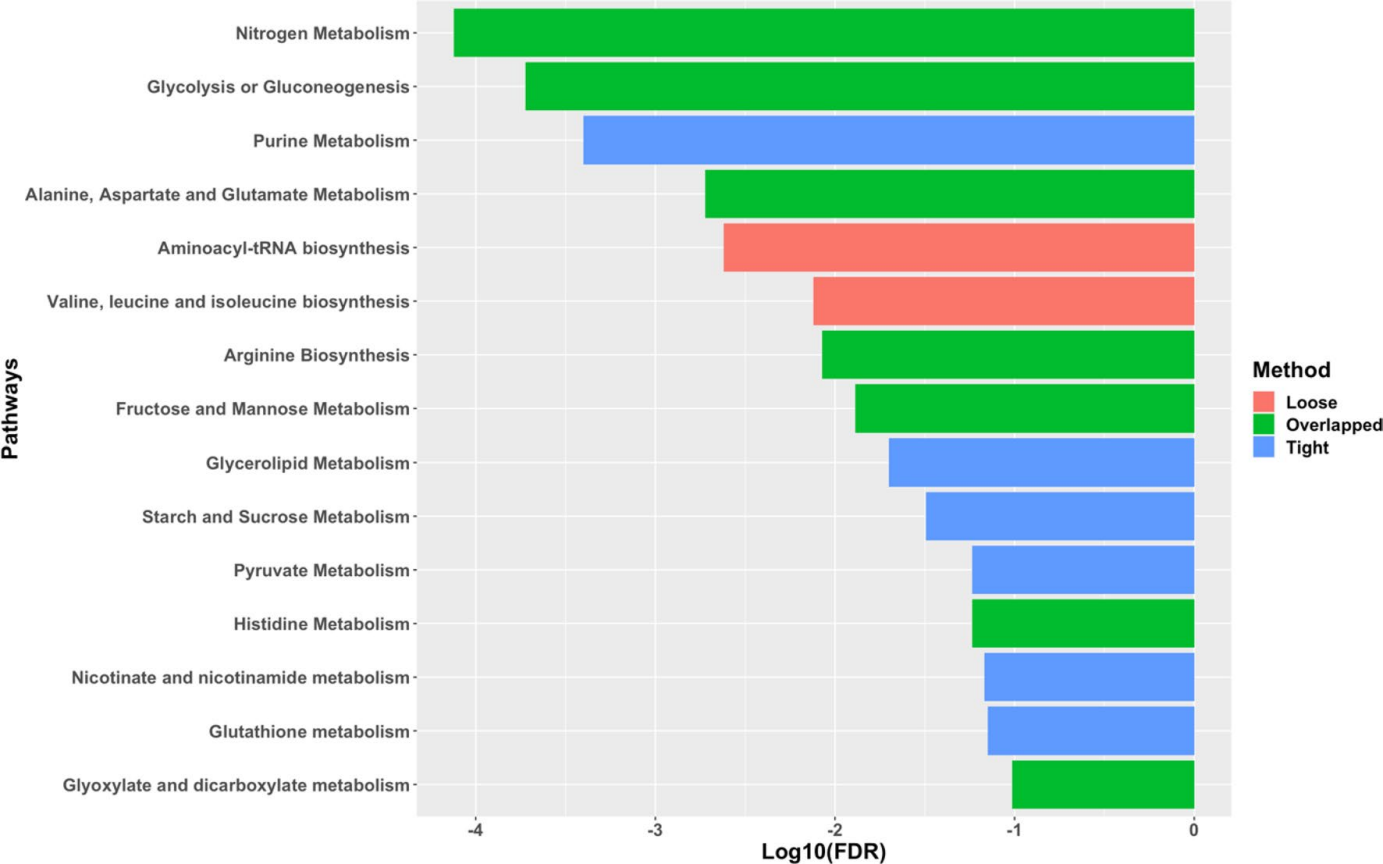
Biological pathways identified by Joint Pathway Analysis of differentially expressed genes and differentially abundant metabolites between WB-affected and unaffected chickens. *FDR* false discovery rate.

In total, 3444 genes were determined to be differentially expressed between WB-affected and unaffected birds with a fold-change (FC) greater than 1.3 and false discovery rate (FDR) threshold smaller than 0.05. Specifically, 1875 were up-regulated and 1569 were down-regulated in WB birds. Through one-way ANOVA, 119 metabolites were found differentially abundant between WB affected and unaffected p. major muscles (FDR < 0.05), among which 90 had higher concentration in WB affected samples. A less stringent FDR threshold value (0.1) was applied for joint pathway analysis in order to include more informative pathways. As a result, 7 out of 9 pathways identified by the loose integration method were overlapped with those selected by the tight integration method, suggesting a rather balanced power between transcriptomic and metabolic profiles in identification of significant features (Fig. 1). Notably, many of the identified pathways are related to amino acid, lipid and energy metabolism. The DEGs pertinent to the identified pathways are reported in Table 1.

**Table 1:** Subset of differentially expressed genes in energy metabolism and peroxisome biosynthesis.

Gene Name	Pathway	Log2FC
Carnosine synthase 1 (CARNS1)	Histidine metabolism	↓2.8
Aconitase 1 (ACO1)	TCA cycle	↑0.5
Isocitrate dehydrogenase (nad(+)) 3 catalytic subunit (IDH3A, IDH3B)	TCA cycle	↓0.8, ↓0.7
Oxoglutarate dehydrogenase (OGDH)	TCA cycle	↓0.8
Succinate-CoA ligase GDP/ADP-forming subunit alpha (SUCLG1)	TCA cycle	↓0.8
Succinate dehydrogenase complex flavoprotein subunit (SDHA, SDHC)	TCA cycle	↓0.8, ↓0.5
Malate dehydrogenase 2 (MDH2)	TCA cycle	↓0.6
Lactate dehydrogenase (LDHA, LDHB, LDHD)	Pyruvate metabolism	↓1.2, ↑1.9, ↓1
d-Aspartate oxidase (DDO)	Ala, Asp, Glu metabolism*	↓1.4
Glutamic-oxaloacetic transaminase (GOT1, GOT2)	Ala, Asp, Glu metabolism	↓0.7, ↓0.8
Asparagine synthetase (ASNS)	Ala, Asp, Glu metabolism	↑2.1
Aspartoacylase (ASPA)	Ala, Asp, Glu metabolism	↓1.4
*N*-Acetyltransferase 8 like (NAT8L)	Ala, Asp, Glu metabolism	↑1.3
Ribosomal modification protein rimk like family member (RIMKLB)	Ala, Asp, Glu metabolism	↑0.7
Glutamate-ammonia ligase (GLUL)	Ala, Asp, Glu metabolism	↓1.5
Glutaminase 2 (GLS2)	Ala, Asp, Glu metabolism	↓1.9
CD36 molecule (CD36)	Fatty acid metabolism	↓0.7
Malonyl-CoA decarboxylase (MLYCD)	Fatty acid metabolism	↓0.7
Carnitine palmitoyltransferase (CPT1A, CPT2)	Fatty acid metabolism	↓0.8, ↑0.6
Acyl-CoA synthetase long chain family member 4 (ACSL4)	Fatty acid metabolism	↓0.5
Solute carrier family 16 member 1 (SLC16A1)	Fatty acid metabolism	↓0.7
3-Oxoacid CoA-transferase 1 (OXCT1)	Fatty acid metabolism	↓0.9
Uncoupling protein 3 (UCP3)	Fatty acid metabolism	↓1.8
Lipin-1 (LPIN1)	Glycerolipid metabolism	↓0.5
Adipose triglyceride lipase (PNPLA2)	Glycerolipid metabolism	↓0.6
Adiponutrin (PNPLA3)	Glycerolipid metabolism	↓0.8
Diacylglycerol o-acyltransferase 2 (DGAT2)	Glycerolipid metabolism	↓1.9
Acyl-CoA wax alcohol acyltransferase 1 (AWAT1)	Glycerolipid metabolism	↓1.7
Hypoxia inducible factor 1 subunit alpha (HIF1A)	Hypoxia	↑0.4
RXR alpha (RXRA)	Hypoxia	↓1.4
Forkhead box o1 (FOXO1)	Hypoxia	↓1.3
Glycerone-phosphate O-acyltransferase (GNPAT)	Ether lipid synthesis	↓0.6
Acyl-CoA reductase (FAR1)	Ether lipid synthesis	↓0.6
Acyl-CoA oxidase 2 (ACOX2)	Fatty acid metabolism	↓0.5
Phytanoyl-CoA 2-Hydroxylase (PHYH)	Fatty acid metabolism	↓0.8
Peroxisome biogenesis factor 1 (PEX1)	Peroxisome biosynthesis	↓0.5
Peroxisome biogenesis factor 5 (PEX5)	Peroxisome biosynthesis	↓0.5
Peroxisome biogenesis factor 7 (PEX7)	Peroxisome biosynthesis	↓0.7
Peroxisome biogenesis factor 10 (PEX10)	Peroxisome biosynthesis	↓0.5

*Alanine, aspartate and glutamate metabolism.

### Weighted gene co-expression network analysis

**Figure 2:**
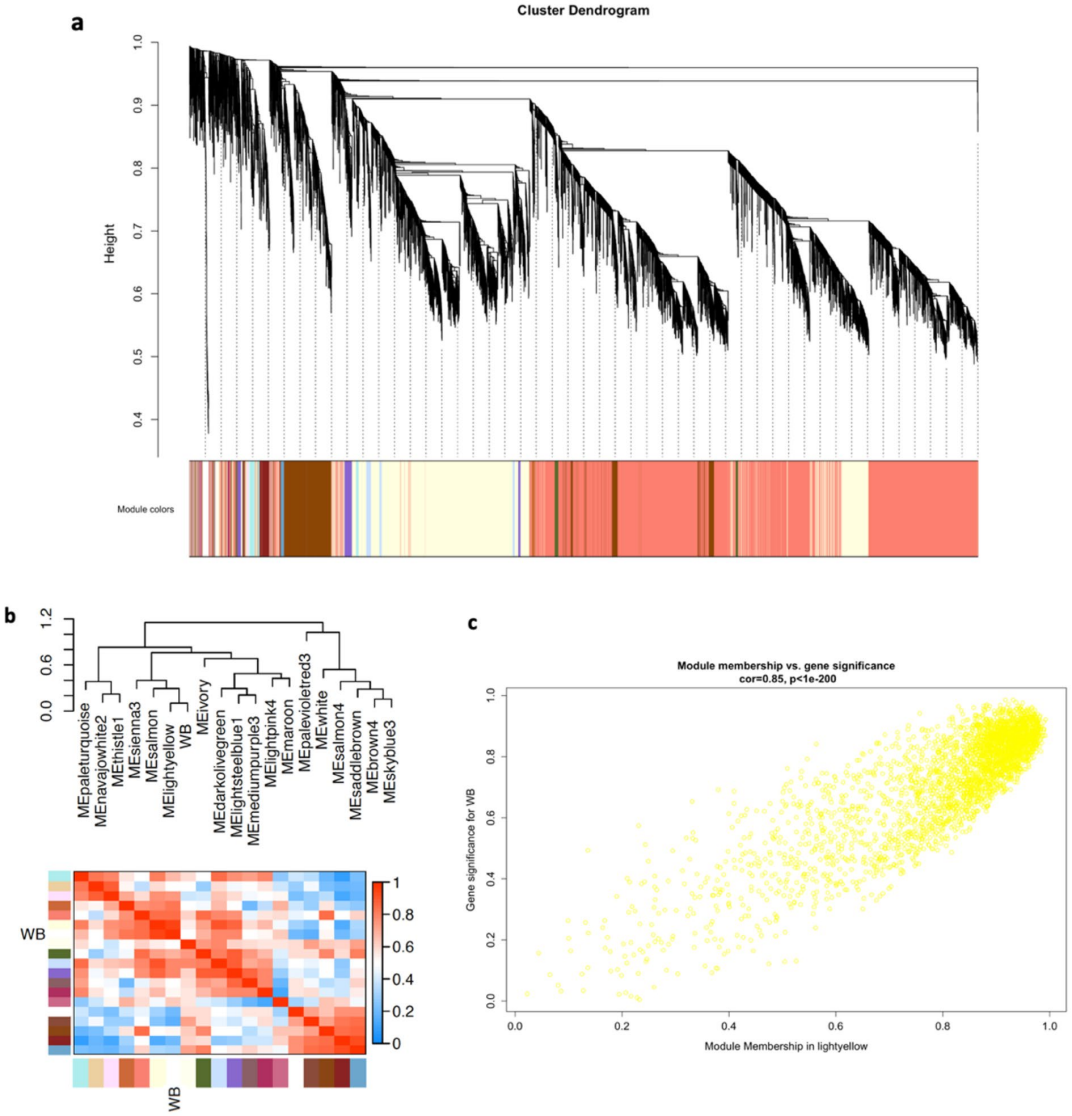
Weighted gene co-expression network analysis of combined RNA-seq and metabolomics data. a. Hierarchical cluster tree of co-expression modules; b. Eigengene network showing relationships among modules and wooden breast myopathy (WB); c. Scatterplots of gene significance (GS) for WB versus module membership (MM) in the lightyellow module. This figure was generated in R v3.5.2 (https://www.R-project.org/) using package WGCNA v1.70-3 (http://horvath.genetics.ucla.edu/html/CoexpressionNetwork/Rpackages/WGCNA/).

Given the scale-free topology index being lower than 0.8 and the high connectivity (Figure S4) between features, the results indicated the existence of an overall strong profile driving the divergence between WB affected and unaffected broilers. In total, WGCNA yielded 19 modules (Fig. 2a), but only the lightyellow module was selected for further analysis based on its positive and significant correlation of 0.9 with WB (Fig. 2b, Fig. S3). Overall, 2842 genes and 172 metabolites were included in this module.

Module membership (MM) is used to quantify the distance between a feature and module eigenvalue based on their correlation, while gene significance (GS) is defined as the correlation between a feature and external traits. Features with both highest MM and GS values were considered as biologically interesting candidates for studying WB^27^. MM and GS in lightyellow module had a strong positive correlation (Fig. 2c), indicating that those highly connected features in this module were also highly correlated with the WB disease. Therefore, intramodular hub features were selected using a more stringent criteria, with both GS and MM > 0.8, as well as falling in the top 10% ranked connectivity. In total, 301 hub features were identified, among which 295 were genes and 6 were metabolites. Top 10 hub features with the highest connectivity were further distinguished (Table 2). Notably, the sign of MM and GS is consistent with the direction of differential abundance of a feature (i.e., if a feature has a positive sign, it is also significantly more abundant in affected chickens compared with unaffected chickens). Interestingly, these top hub features had a high overlap of 2593 connected genes. Functional analysis on these overlapped genes revealed many signaling pathways including focal adhesion, phagosome, regulation of actin cytoskeleton, ECM-receptor interaction, FoxO signaling pathway, apoptosis and so on.

**Table 2:** Module membership and gene significance of the top 10 hub features in lightyellow module of weighted gene co-expression network analysis in wooden breast myopathy (WB) muscle samples.

Feature	MM	GS
Brain Abundant Membrane Attached Signal Protein 1 (BASP1)	0.99	0.92
Zyxin (ZYX)	0.99	0.87
Actinin Alpha 1 (ACTN1)	0.99	0.89
Frizzled Class Receptor 1 (FZD1)	0.99	0.91
Complement C1r (C1R)	0.99	0.89
Inosine 5′-monophosphate (IMP)	− 0.97	− 0.89
Coenzyme Q9 (COQ9)	− 0.96	− 0.87
Carnosine Synthase 1 (CARNS1)	− 0.96	− 0.89
Glutamic-Oxaloacetic Transaminase 2 (GOT2)	− 0.95	− 0.87
Phosphofructokinase, Muscle (PFKM)	− 0.95	− 0.90

*MM* module membership, *GS* gene significance.

## Discussion

In accordance with previous research on WB^6,7^, closer examination of the pathways obtained above revealed a rather broad range of biological perturbations in the WB p. major muscle, from mitochondrial functionality, energy metabolism to hypoxia and oxidative stress. The following sections discuss these metabolic pathways in play in more detail.

### Histidine metabolism

**Figure 3:**
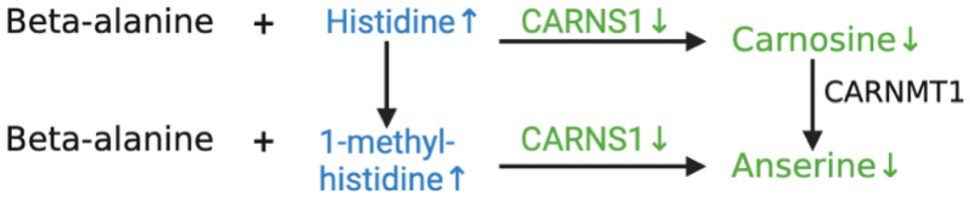
Reduced levels of carnosine and anserine in wooden breast affected chickens, likely due to down-regulation of carnosine synthase 1 (CARNS1). Different colors indicate higher (blue), lower (green) and not statistically different (black) abundance of a metabolite or gene transcripts in wooden breast affected p. major muscle. This figure was created with BioRender.com (http://biorender.com).

Histidine is an essential amino acid in chickens as a limiting factor for carnosine and anserine synthesis^28^. As histidine related compounds, carnosine and anserine are important for their roles in scavenging reactive oxygen species (ROS) and buffering intracellular pH^28^. In accordance with the previous study using the same dataset^7^, histidine and related metabolites 1-methyl-histidine and 3-methyl-histidine were significantly higher in WB affected breast muscle, whereas carnosine and anserine were found significantly lower (Fig. 3; Table S5). Our integrative analysis identified a potential mechanism for this decrease, attributed to the down-regulation of carnosine synthase 1 (CARNS1) in WB affected birds. Since CARNS1 synthesizes carnosine and anserine from histidine, 1-methyl-histidine and beta-alanine^29,30^, decreased carnosine levels could result in impaired antioxidant capacity and hence the widely reported oxidative stress in WB^6,7,31^. Khumpeerawat and colleagues reported moderate correlation between CARNS1 levels and carnosine content in chickens, with both having an inverse relationship with bird age^29^. In addition, 3-methyl-histidine was considered a marker for myofibrillar breakdown and found to have high prediction accuracy for WB^32^. Dietary supplementation of beta-alanine and histidine led to increased carnosine levels synthesized in both slow and fast-growing chickens^30,33^, which in turn may potentially increase antioxidant capacity^30,33,34^. However, such increase was not observed in anserine^29,33^, suggesting that anserine synthesis in chicken pectoral muscle relies rather heavily on the methylation of carnosine from *S*-adenosylmethionine by carnosine-*N*-methyltransferase (CARNMT1) instead of 1-methyl-histidine^35^. While methionine content was significantly higher in WB affected muscle, the enzyme in charge of the production of *S*-adenosylmethionine, methionine adenosyltransferase (MAT2A), was down-regulated by a FC of 1.7. This could explain why increased levels of carnosine failed to boost anserine levels in chickens. That said, it is worthwhile for future studies to confirm the scarcity of *S*-adenosylmethionine and whether the reinstated level of carnosine and anserine could ameliorate WB prevalence and severity in broilers.

### Remodeling of energy metabolism

**Figure 4:**
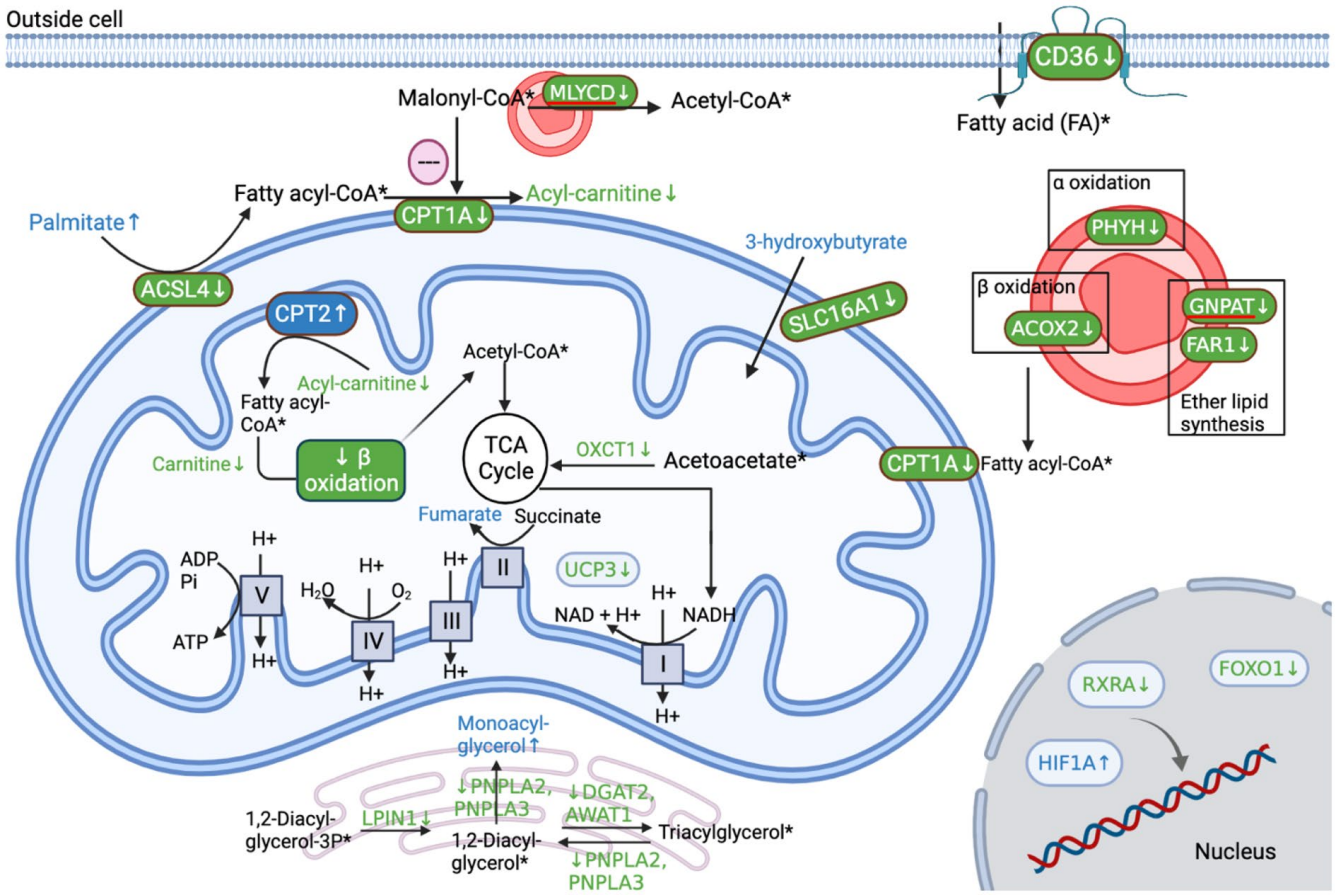
Proposed schematic representation of remodeled energy metabolism in WB p. major muscles. Colors indicate higher (blue), lower (green) and not statistically different (black) abundance of a metabolite or gene transcripts in wooden breast affected p. major muscles in broiler chickens. *Not detected in metabolome data. *CD36* CD36 molecule, *MLYCD* Malonyl-CoA decarboxylase, *CPT1A, CPT2* Carnitine palmitoyltransferase, *ACSL4* Acyl-CoA synthetase long chain family member 4, *SLC16A1 Solute* carrier family 16 member 1, *OXCT1* 3-oxoacid CoA-transferase 1, *UCP3* Uncoupling protein 3, *LPIN1* Lipin-1, *PNPLA2* Adipose triglyceride lipase, *PNPLA3* Adiponutrin, *DGAT2* Diacylglycerol o-acyltransferase 2, *AWAT1* Acyl-CoA wax alcohol acyltransferase 1, *HIF1A* Hypoxia inducible factor 1 subunit alpha, *RXRA* RXR alpha, *FOXO1* Forkhead box o1, *GNPAT* Glycerone-phosphate O-acyltransferase, *FAR1* Acyl-CoA reductase, *ACOX2* Acyl-CoA oxidase 2, *PHYH* Phytanoyl-CoA 2-Hydroxylase. This figure was created with BioRender.com (http://biorender.com).

As shown in Fig. 1, our Joint Pathway Analysis identified several pathways involved in energy metabolism including glycolysis or gluconeogenesis; alanine, aspartate and glutamate metabolism; glycerolipid metabolism; pyruvate metabolism and histidine metabolism. WB tissues manifested accumulation of monoacylglycerols 2-linoleoylglycerol, 1-palmitoylglycerol and glycerol-3-phosphate (Table S5), which is consistent with decreased expression of genes involved in TAG synthesis [diacylglycerol o-acyltransferase 2 (DGAT2), acyl-CoA wax alcohol acyltransferase 1 (AWAT1)] and degradation [adipose triglyceride lipase (PNPLA2), adiponutrin (PNPLA3)] in affected tissues (Fig. 4). Besides the potential reduction in TAG metabolism, the affected muscles also exhibited down-regulation of key enzymes participating in mitochondrial FA oxidation (Fig. 4), including carnitine palmitoyltransferase I (CPT1A), malonyl-CoA decarboxylase (MLYCD), uncoupling protein 3 (UCP3), acyl-CoA synthetase long chain family member 4 (ACSL4) and FA translocase (CD36). Downregulation of these genes agreed with the significantly lower levels of their substrates, carnitines and acyl-carnitines, as well as higher levels of palmitate (Table S5). It is noteworthy to mention that broilers of 2–3 weeks of age with early stage WB exhibited upregulation of CD36 and some other lipid genes^13–15^. Similarly, at 7 weeks of age, expression of a key enzyme in lipid metabolism, lipoprotein lipase (LPL), was higher in moderately affected chickens when compared with chickens severely affected with WB^36^. These findings suggested dysregulated and even shifting role of lipid metabolism in different stages of WB.

Additional evidence indicating abnormal energy metabolism in WB is the build-up of 3-hydroxybutyrate in affected tissues (Table S5; Fig. 4). This accumulation could be caused by the down-regulation of solute carrier family 16 member 1 (SLC16A1; FC of 1.7), denoting less transporters available for the entry of 3-hydroxybutyrate into mitochondria^37^, and hence restricting its use as a fuel for muscle. In addition to this ketone body, the use of acetoacetate for energy production may also be restricted in WB, as indicated by the down-regulation of an essential enzyme in ketolysis, 3-oxoacid CoA-transferase 1 (OXCT1; Fig. 4), which catalyzes the production of acetyl-CoA for the TCA cycle from acetoacetate^37^.

Differential expression of numerous transcription factors (Fig. 4) relevant to energy metabolism were noted in WB affected muscle, hypoxia inducible factor 1 subunit alpha (HIF1A), RXR alpha (RXRA) and forkhead box o1 (FOXO1). Under hypoxia, diminished FOXO1 and RXRA level suppress expression of their target genes such as UCP3, CD36 and ACSL4 (Fig. 4), resulting in declined FA uptake and oxidation^38,39^.

Subsequently, oxidative phosphorylation may have also been hindered, as shown by related DEGs (Table 3) and a significantly lower level of phosphate (Table S5), which is a cytosolic regulator of this process through the cellular energy state feedback^40,41^. The down-regulation of ATP synthase mitochondrial F1 complex assembly factor 1 (ATPAF1) is associated with a lower respiratory capacity and degenerated mitochondria because of impaired ATP synthase assembly^4,42^. Overall, these metabolic and transcriptomic variations implied a negative energy balance due to impaired mitochondrial capacity in WB affected p. major muscles.

**Table 3:** Differentially expressed genes involved in oxidative phosphorylation.

Complex	Gene symbol	
	Up-regulated	Down-regulated
I	–	ND4L, ND4, ND3, ND1, NDUFS8, NDUFS7, NDUFS6, NDUFS1, NDUFA7, NDUFA11, NDUFA12, NDUFB10
II	–	SDHC, SDHA
III	–	UQCRFS1, CYTB, UQCRC2, UQCRQ
IV	COX6A1, COX7A2	–
V	ATP6V1C2, ATP6V1A, ATP6V0D2	ATPAF1, ATP5L, ATP6, ATP6V0A1

**Figure 5:**
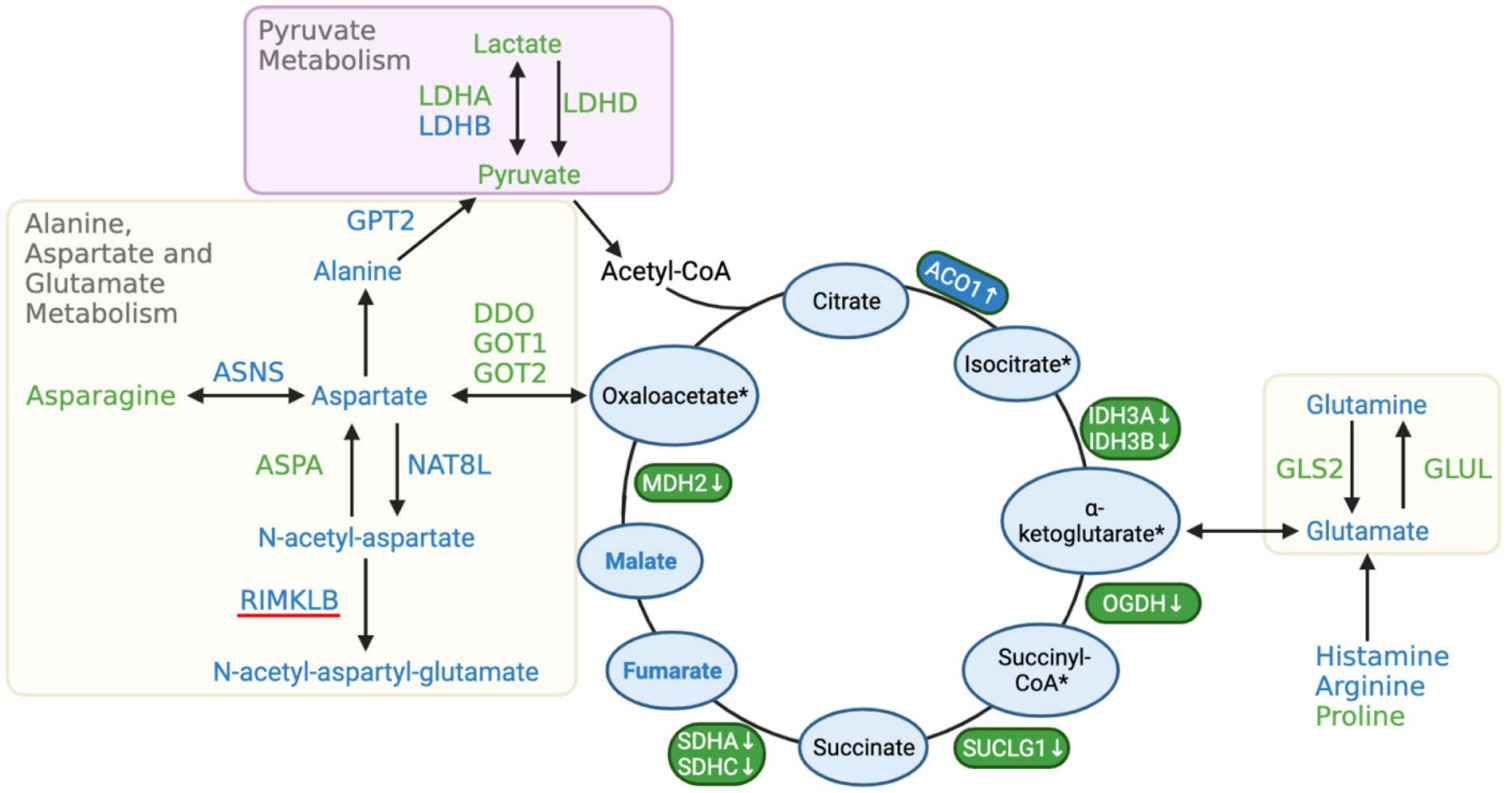
Schematic representation of altered TCA cycle and increase in amino acids involved in anaplerosis in breast muscles affected with wooden breast myopathy. Colors indicate higher (blue), lower (green) and not statistically different (black) abundance of a metabolite or gene transcripts in wooden breast affected p. major muscles in broiler chickens. * Not detected in metabolome data. *ACO1* Aconitase 1, *nad(+)*Isocitrate dehydrogenase, *IDH3A, IDH3B* 3 catalytic subunit, *OGDH* Oxoglutarate dehydrogenase, *SUCLG1* Succinate-CoA ligase GDP/ADP-forming subunit alpha, *SDHA, SDHC* Succinate dehydrogenase complex flavoprotein subunit, *MDH2* Malate dehydrogenase 2, *LDHA, LDHB*, *LDHD* Lactate dehydrogenase, *DDO* D-aspartate oxidase, *GOT1, GOT2* Glutamic-oxaloacetic transaminase. *ASNS* Asparagine synthetase, *ASPA* Aspartoacylase, *NAT8L* N-acetyltransferase 8 like, *RIMKLB* Ribosomal modification protein rimk like family member, *GLUL* Glutamate-ammonia ligase, *GLS2* Glutaminase 2. This figure was created with BioRender.com (http://biorender.com).

In response to a negative energy balance, normally, “starved” myocytes may increase the rate of amino acid catabolism for the purpose of refilling the pools of intermediates in the TCA cycle, via a process termed anaplerosis^3,43^. However, this may not be occurring in WB affected broiler chickens (Fig. 5). Although amino acids serving as gluconeogenic and ketogenic precursors^43^ were significantly higher in WB tissue, down-regulation of D-aspartate oxidase (DDO) and glutamic–oxaloacetic transaminase (GOT1, GOT2) suggested an obstructed path to replenish the TCA cycle. Additionally, GOT1 was identified as a candidate gene for WB in a genome-wide association study^44^, further corroborating an attenuated energy metabolism from genetic basis. Proline dehydrogenase (PRODH) is the rate limiting enzyme for proline degradation and catalyzes the electron transfer from proline to flavine adenine dinucleotide (FAD) before passing on to cytochrome c in electron transport chain to generate ATP^45^. Accordingly, the upregulation of PRODH by a FC of 3.3, higher levels of FAD and lower levels of proline (Table S5) implied ATP shortage, even nutrient and inflammatory stress in the WB p. major muscle^45^. One potential explanation for this malfunctioned anaplerosis is the modern broilers’ genetic predisposition for muscular hypertrophy, driving most of its available amino acids towards protein synthesis.

### Peroxisomal function

Peroxisomes are unique organelles indispensable to various vital metabolic pathways including FA alpha- and beta-oxidation, ROS metabolism and etherphospholipid synthesis^46,47^. More precisely, other subcellular organelles rely on the functional interplay with peroxisomes to fulfill their role in metabolism. For instance, very-long-chain FAs need to undergo stepwise shortening within peroxisomes before they can be shuttled into mitochondria for full oxidation^46^. In addition to compromised mitochondrial functions, our study also suggests impaired peroxisomal function in WB muscle.

Glycerone-phosphate O-acyltransferase (GNPAT) and acyl-CoA reductase (FAR1) are responsible for the synthesis of dihydroxyacetone phosphate and long-chain alcohols, respectively, before the formation of ether bond between them^48^. Therefore, the down-regulation of these two enzymes (Table 1) could hamper etherphospholipid synthesis by reducing the substrate level. In humans, the deficiency of GNPAT and/or FAR1 is linked to plasmalogen, or cell membrane glycerophospholipids, deprivation due to insufficient etherphospholipid synthesis^46^. Furthermore, significantly higher levels of glycerophosphorylcholine (GPC) and glycerophosphoethanolamine (GPE) in WB affected muscles (Table S5) could be indicating lower plasmalogen biosynthesis as well as turnover^49^. Given that plasmalogens are essential components of cell and subcellular membranes^49^, its reduction could result in more frequent membrane damage, and hence loss of cellular integrity. Considering that membrane biosynthesis increases as muscles grow^50^, a potential reduction in plasmalogens and the ensuing membrane instability could be even more problematic in the face of the increased hypertrophy in fast-growing broiler chickens.

Additionally, it is likely that peroxisome biogenesis was diminished in WB chickens, as shown by the down-regulation of peroxisome biogenesis factors (PEX1, PEX5, PEX7, PEX10) by an average FC of 1.5 (Table 1). Specifically, cytoplasmic receptors PEX5 and PEX7 recognize and bind to proteins containing peroxisome targeting signal (PTS)^51^, which are then imported into peroxisomes by a complex consisting of PEX2, PEX10 and PEX12^52^. These PTS matrix protein receptors exit peroxisomes to be recycled back to the cytosol with the help from PEX1, PEX6 and integral membrane protein PEX26^52^. An aberrant mitochondrial ultrastructure and respiratory chain activity were observed in PEX5 knockout mice, resulting from defective peroxisome biogenesis^53^.

Considering the supportive role of peroxisomes in metabolism, we hypothesize that albeit in a chronic manner, a potential decrease in their biogenesis in WB could eventually be detrimental, due to an increase in ROS levels and mitochondrial defects^51,53^. A study in *Drosophila* suggested a linkage between selective peroxisome loss and muscle function and physiology by disrupting energy metabolism^51^. Consequently, it is worthwhile to obtain a deeper understanding of the particular role played by peroxisomes in WB disease progression.

### Cellular signaling

Functional analysis of the interconnected network surrounding the hub features implied differences in cell–cell communication between WB affected and unaffected chickens, which are in line with results from a recent study on 7-week-old WB affected broilers^54^. Focal adhesions are the linking bridges between cells and extracellular matrix (ECM), where integrin, proteoglycan and actin cytoskeleton mediate mechanical and biochemical signaling^55^. In skeletal muscles, the cell-ECM crosstalk is not only vital for muscle contraction but can also modulate myogenesis and muscle repair^55^. It is likely that myofibrillar changes in the WB muscle happened concurrently with alterations in focal adhesion, as shown by DEGs in ECM-receptor interaction, cytokine-cytokine receptor interaction and Wnt signaling pathways (Tables S2–S4). Specifically, actinin alpha 1 (ACTN1) is responsible for crosslinking integrin to actin filaments in the cytoskeleton, and zyxin (ZYX) is recruited to newly formed focal complexes to stabilize membrane protrusion^56,57^. In the meantime, calpain (CAPN2) cleaves these proteins within focal adhesions to mediate their overall turnover and cell migration^57^. The ECM relevant DEGs such as integrin subunit alpha and beta and collagens (Table S2) also suggested aberrant ECM composition and interactions. Additionally, ZYX and ACTN1 are associated with cytoskeletal remodeling in vascular smooth muscle cells and vessel stiffness^58,59^. The upregulated ACTN1 and collagen type I alpha 1 chain (COL1A1) observed in WB chickens were concordant with changes in vascular smooth muscle cells during vascular regression^59^, which could possibly contribute to WB’s signature lesion phlebitis^4^.

One of the top hub features in WGCNA is inosine 5′-monophosphate (IMP), which can mediate diverse processes including inflammation as an extracellular signaling molecule^60^. Although the role of IMP in cellular signaling remains unclear, its importance was suggested by the high connectivity between IMP and other features in the network. Compared with unaffected samples, WB affected muscles showed a significant difference in the levels of IMP, adenosine 5′-monophosphate (AMP), guanosine 5′-monophosphate (GMP) and purine bases except for inosine (Table S5). The significantly lower level of IMP and AMP, as well as the down-regulation of AMP deaminase 1 (AMPD1) in WB tissues may even impose a negative effect in purine biosynthesis, as it is likely that the replenishment of IMP from AMP was impeded^60^. Moreover, catabolic product hypoxanthine from purine metabolism was linked to muscle fatigue and depletion of energy substrates^61^. However, unlike in mice^61^, the accumulation of hypoxanthine in affected broilers was not accompanied with enhanced glycolysis, mitochondrial biogenesis or oxidative phosphorylation, as shown by down-regulated DEGs (Table 3, Table S1), perhaps because of a genetic predisposition to diverting resources toward protein metabolism.

## Conclusion

The integration of transcriptomics and metabolomics enabled us to study pathways relevant to the WB progression by possibly linking genes and their metabolites within biological pathways. In particular, we showed a potential mechanism for impaired antioxidant capacity in WB, which involves the down-regulation of CARNS1, resulting in the depletion of its antioxidant products carnosine and anserine. Furthermore, this study hypothesized that the buildup of lipid intermediates presumably due to down-regulated lipid genes leads to lipid toxicity^62^ and mitochondrial dysfunction in WB. Our results also suggest an increased use of some energy precursors for protein synthesis in WB, so it may be of interest to study the mechanism behind the switch between energy metabolism and anabolic muscle growth in WB. Correlated genes related to focal adhesion indicated that intercellular signaling may play a role in regulating, even altering, different biological pathways during WB development.

## Supplementary Information

Supplementary Information.

## Data Availability

RNA-seq data used are available at the NCBI Sequence Read Archive via BioProject accession number: PRJNA563347. Numeric representations of the spectral entries for the metabolites identified in our sample set are provided as a supplementary file in Abasht et al.^7^.

## References

[1] Caplen G (2012). Kinematic analysis quantifies gait abnormalities associated with lameness in broiler chickens and identifies evolutionary gait differences. PLoS One.

[2] Kuttappan VA, Hargis BM, Owens CM (2016). White striping and woody breast myopathies in the modern poultry industry: A review. Poult. Sci..

[3] Sihvo H-K, Immonen K, Puolanne E (2013). Myodegeneration with fibrosis and regeneration in the pectoralis major muscle of broilers. Vet. Pathol..

[4] Papah MB, Brannick EM, Schmidt CJ, Abasht B (2017). Evidence and role of phlebitis and lipid infiltration in the onset and pathogenesis of Wooden Breast Disease in modern broiler chickens. Avian Pathol..

[5] Hubert SM, Athrey G (2020). Energy metabolism and sources of oxidative stress in wooden breast—a review. F1000Res.

[6] Mutryn MF, Brannick EM, Fu W, Lee WR, Abasht B (2015). Characterization of a novel chicken muscle disorder through differential gene expression and pathway analysis using RNA-sequencing. BMC Genom..

[7] Abasht B, Mutryn MF, Michalek RD, Lee WR (2016). Oxidative stress and metabolic perturbations in Wooden Breast disorder in chickens. PLoS One.

[8] Marie Hubert S, Williams TJ, Athrey G (2018). Insights into the molecular basis of wooden breast based on comparative analysis of fast- and slow-growth broilers. biorxiv.

[9] Tallentire CW, Leinonen I, Kyriazakis I (2016). Breeding for efficiency in the broiler chicken: A review. Agron. Sustain. Dev..

[10] Kong B-W (2017). RNA sequencing for global gene expression associated with muscle growth in a single male modern broiler line compared to a foundational Barred Plymouth Rock chicken line. BMC Genom..

[11] Banerjee P, Carmelo VAO, Kadarmideen HN (2020). Integrative analysis of metabolomic and transcriptomic profiles uncovers biological pathways of feed efficiency in pigs. Metabolites.

[12] Connor SC, Hansen MK, Corner A, Smith RF, Ryan TE (2010). Integration of metabolomics and transcriptomics data to aid biomarker discovery in type 2 diabetes. Mol. Biosyst..

[13] Papah MB, Abasht B (2019). Dysregulation of lipid metabolism and appearance of slow myofiber-specific isoforms accompany the development of Wooden Breast myopathy in modern broiler chickens. Sci. Rep..

[14] Papah MB, Brannick EM, Schmidt CJ, Abasht B (2018). Gene expression profiling of the early pathogenesis of wooden breast disease in commercial broiler chickens using RNA-sequencing. PLoS One.

[15] Lake JA, Papah MB, Abasht B (2019). Increased expression of lipid metabolism genes in early stages of wooden breast links myopathy of broilers to metabolic syndrome in humans. Genes (Basel).

[16] “Support from the University of Delaware CBCB Bioinformatics Core Facility and use of the BIOMIX compute cluster was made possible through funding from Delaware INBRE (NIH NIGMS P20 GM103446), the State of Delaware, and the Delaware Biotechnology Institut.”

[17] Andrews, S. FastQC: A Quality Control Tool for High Throughput Sequence Data (2010).

[18] Kim D, Langmead B, Salzberg SL (2015). HISAT: A fast spliced aligner with low memory requirements. Nat. Methods.

[19] Anders S, Pyl PT, Huber W (2015). HTSeq—a Python framework to work with high-throughput sequencing data. Bioinformatics.

[20] Evans AM, DeHaven CD, Barrett T, Mitchell M, Milgram E (2009). Integrated, nontargeted ultrahigh performance liquid chromatography/electrospray ionization tandem mass spectrometry platform for the identification and relative quantification of the small-molecule complement of biological systems. Anal. Chem..

[21] Xia J, Psychogios N, Young N, Wishart DS (2009). MetaboAnalyst: A web server for metabolomic data analysis and interpretation. Nucleic Acids Res..

[22] Ripley BD (2001). The R project in statistical computing. MSOR Connect..

[23] Robinson MD, Oshlack A (2010). A scaling normalization method for differential expression analysis of RNA-seq data. Genome Biol..

[24] Pang Z (2021). MetaboAnalyst 5.0: Narrowing the gap between raw spectra and functional insights. Nucleic Acids Res..

[25] Zhang B, Horvath S (2005). A general framework for weighted gene co-expression network analysis. Stat. Appl. Genet. Mol. Biol..

[26] Langfelder P, Horvath S (2008). WGCNA: An R package for weighted correlation network analysis. BMC Bioinform..

[27] Wang W (2017). Weighted gene co-expression network analysis of expression data of monozygotic twins identifies specific modules and hub genes related to BMI. BMC Genomics.

[28] Moro J, Tome D, Schmidely P, Demersay TC, Azzout-Marniche D (2020). Histidine: A systematic review on metabolism and physiological effects in human and different animal species.. Nutrients.

[29] Khumpeerawat P, Duangjinda M, Phasuk Y (2021). Carnosine content and its association with carnosine-related gene expression in breast meat of Thai native and black-bone chicken. Animals (Basel).

[30] Suwanvichanee C (2022). Effects of β-alanine and L-histidine supplementation on carnosine contents in and quality and secondary structure of proteins in slow-growing Korat chicken meat. Poult. Sci.

[31] Zhang, X. *et al.* Proteomic characterization of normal and woody breast meat from broilers of five genetic strains. *Meat Muscle Biol*. **4**, (2020).

[32] Lake JA (2022). Identification of circulating metabolites associated with wooden breast and white striping. PLoS One.

[33] Kopec W (2020). Antioxidative characteristics of chicken breast meat and blood after diet supplementation with carnosine, L-histidine, and β-alanine. Antioxidants (Basel).

[34] Hu X (2009). Effect of carnosine on growth performance, carcass characteristics, meat quality and oxidative stability in broiler chickens. J. Poult. Sci.

[35] McManus IR (1962). Enzymatic synthesis of anserine in skeletal muscle by N-methylation of carnosine. J. Biol. Chem.

[36] Abasht B, Papah MB, Qiu J (2021). Evidence of vascular endothelial dysfunction in Wooden Breast disorder in chickens: Insights through gene expression analysis, ultra-structural evaluation and supervised machine learning methods. PLoS One.

[37] Evans M, Cogan KE, Egan B (2017). Metabolism of ketone bodies during exercise and training: physiological basis for exogenous supplementation: Ketone bodies and exercise. J. Physiol.

[38] Szanto A (2004). Retinoid X receptors: X-ploring their (patho)physiological functions. Cell Death Differ..

[39] Bastie CC (2005). FoxO1 stimulates fatty acid uptake and oxidation in muscle cells through CD36-dependent and -independent mechanisms. J. Biol. Chem..

[40] Bose S, French S, Evans FJ, Joubert F, Balaban RS (2003). Metabolic network control of oxidative phosphorylation: Multiple rolevascs of inorganic phosphate. J. Biol. Chem.

[41] Wilson DF (2017). Oxidative phosphorylation: regulation and role in cellular and tissue metabolism: Oxidative phosphorylation: role in cell and tissue metabolism. J. Physiol.

[42] Zhou Z (2021). ATPAF1 deficiency impairs ATP synthase assembly and mitochondrial respiration. Mitochondrion.

[43] Owen OE, Kalhan SC, Hanson RW (2002). The key role of anaplerosis and cataplerosis for citric acid cycle function. J. Biol. Chem.

[44] Lake JA, Dekkers JCM, Abasht B (2021). Genetic basis and identification of candidate genes for wooden breast and white striping in commercial broiler chickens. Sci. Rep.

[45] Phang JM, Pandhare J, Liu Y (2008). The metabolism of proline as microenvironmental stress substrate. J. Nutr.

[46] Wanders RJA, Waterham HR, Ferdinandusse S (2015). Metabolic interplay between peroxisomes and other subcellular organelles including mitochondria and the endoplasmic reticulum. Front. Cell Dev. Biol.

[47] Liu J, Lu W, Shi B, Klein S, Su X (2019). Peroxisomal regulation of redox homeostasis and adipocyte metabolism. Redox Biol.

[48] Cheng JB, Russell DW (2004). Mammalian wax biosynthesis. I. Identification of two fatty acyl-Coenzyme A reductases with different substrate specificities and tissue distributions.. J. Biol. Chem.

[49] Braverman NE, Moser AB (2012). Functions of plasmalogen lipids in health and disease. Biochim. Biophys. Acta.

[50] Carta G, Murru E, Banni S, Manca C (2017). Palmitic acid: Physiological role, metabolism and nutritional implications.. Front. Physiol..

[51] Faust JE (2014). Peroxisomes are required for lipid metabolism and muscle function in Drosophila melanogaster. PLoS One.

[52] Steinberg SJ (2006). Peroxisome biogenesis disorders. Biochim. Biophys. Acta.

[53] Martens K (2012). Peroxisome deficient aP2-Pex5 knockout mice display impaired white adipocyte and muscle function concomitant with reduced adrenergic tone. Mol. Genet. Metab.

[54] Malila Y (2021). Insights into transcriptome profiles associated with wooden breast myopathy in broilers slaughtered at the age of 6 or 7 weeks. Front. Physiol.

[55] Csapo R, Gumpenberger M, Wessner B (2020). Skeletal muscle extracellular matrix - what do we know about its composition, regulation, and physiological roles? A narrative review. Front. Physiol.

[56] Hsu CP, Moghadaszadeh B, Hartwig JH, Beggs AH (2018). Sarcomeric and nonmuscle α-actinin isoforms exhibit differential dynamics at skeletal muscle Z-lines. Cytoskeleton (Hoboken).

[57] Wozniak MA, Modzelewska K, Kwong L, Keely PJ (2004). Focal adhesion regulation of cell behavior. Biochim. Biophys. Acta.

[58] Suphamungmee W, Lehman W, Morgan KG (2022). Functional remodeling of the contractile smooth muscle cell cortex, a provocative concept, supported by direct visualization of cortical remodeling. Biology (Basel).

[59] Rodriguez, D. *et al.* Vascular Aging in the Invertebrate Chordate, Botryllus schlosseri. *Front. Mol. Biosci.* **8**, 626827 (2021). 10.3389/fmolb.2021.626827 PMC8060491 33898513

[60] Lovászi, M. *et al.* Inosine monophosphate and inosine differentially regulate endotoxemia and bacterial sepsis. *FASEB J.* **35**, e21935 (2021). 10.1096/fj.202100862R PMC9812230 34591327

[61] Yin, C. *et al.* Hypoxanthine induces muscular ATP depletion and fatigue via UCP2. *Front. Physiol.* **12**, 647743 (2021). 10.3389/fphys.2021.647743 PMC7966526 33746782

[62] Listenberger, L. L. *et al.* Triglyceride accumulation protects against fatty acid-induced lipotoxicity. *Proc. Natl. Acad. Sci. U. S. A.* **100**, 3077–3082 (2003). 10.1073/pnas.0630588100 PMC152249 12629214

