# Supplementary material for: Integrative transcriptomic and metabolomic analysis reveals alterations in energy metabolism and mitochondrial functionality in broiler chickens with wooden breast
Source: Sci Rep. 2023 Mar 23;13:4747. doi: 10.1038/s41598-023-31429-7 (PMC10036619; doi:10.1038/s41598-023-31429-7)
Supplement: Supplementary file 1 — Supplementary Information. [file 41598_2023_31429_MOESM1_ESM.docx]

Supplementary information


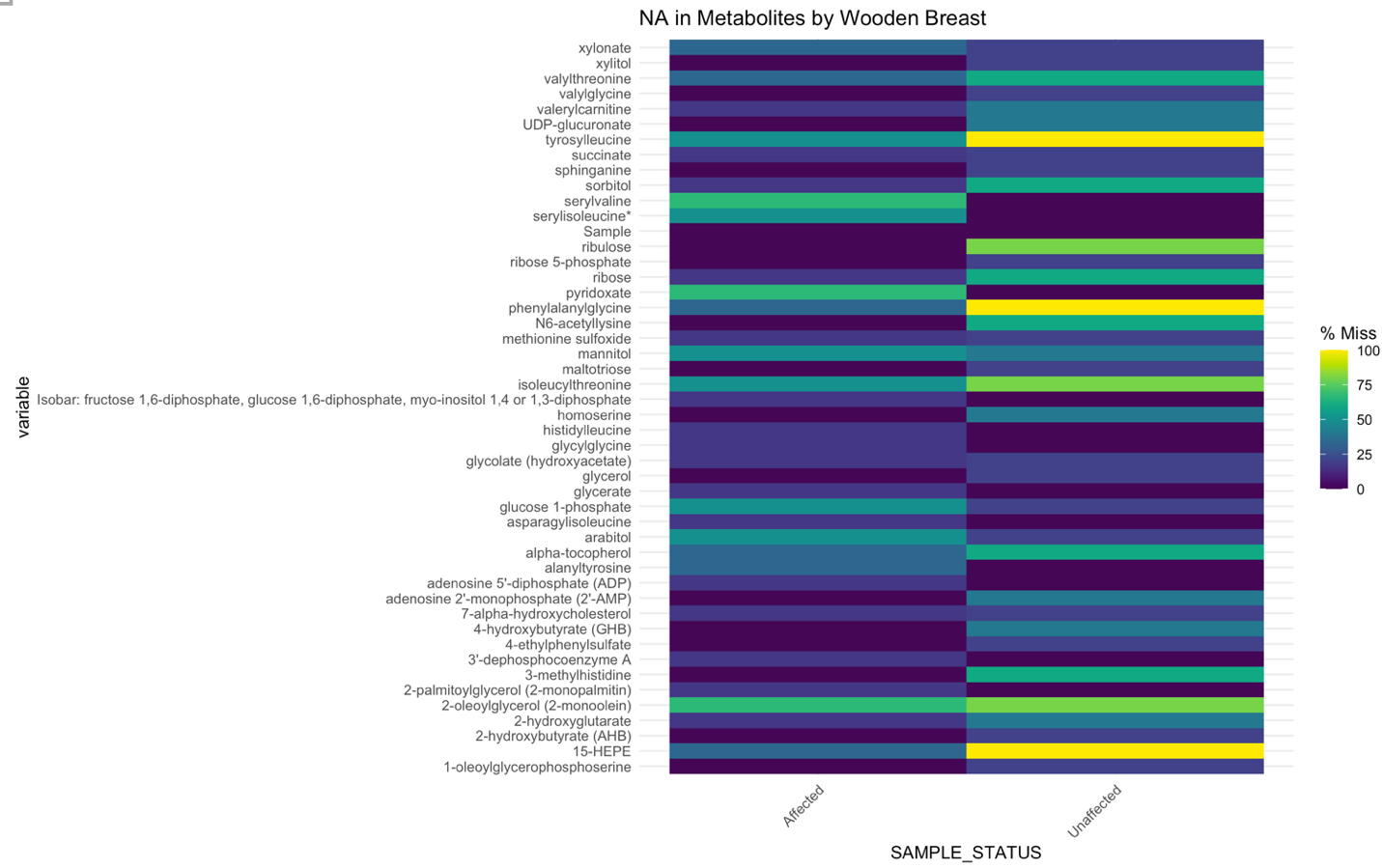


*Figure S1. Missingness of metabolites in obtained metabolomics data in Wooden-Breast-affected and unaffected broiler chickens.*

*
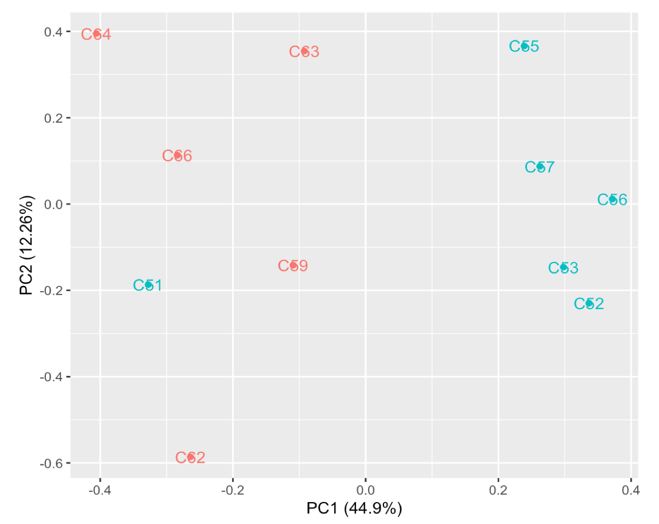

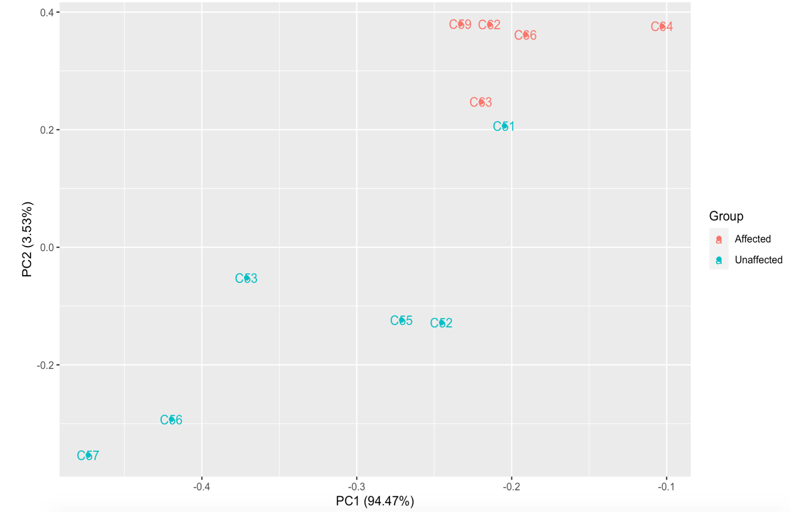
*

*Figure S2: Principal component analysis (PCA) showing reclassified subclinical Wooden Breast sample C51 grouped with Wooden Breast affected pectoralis major muscle samples by metabolomics (left) and RNA-seq (right). This figure was generated in R v3.5.2 (*[*https://www.R-project.org/*](https://www.R-project.org/)*)*

*
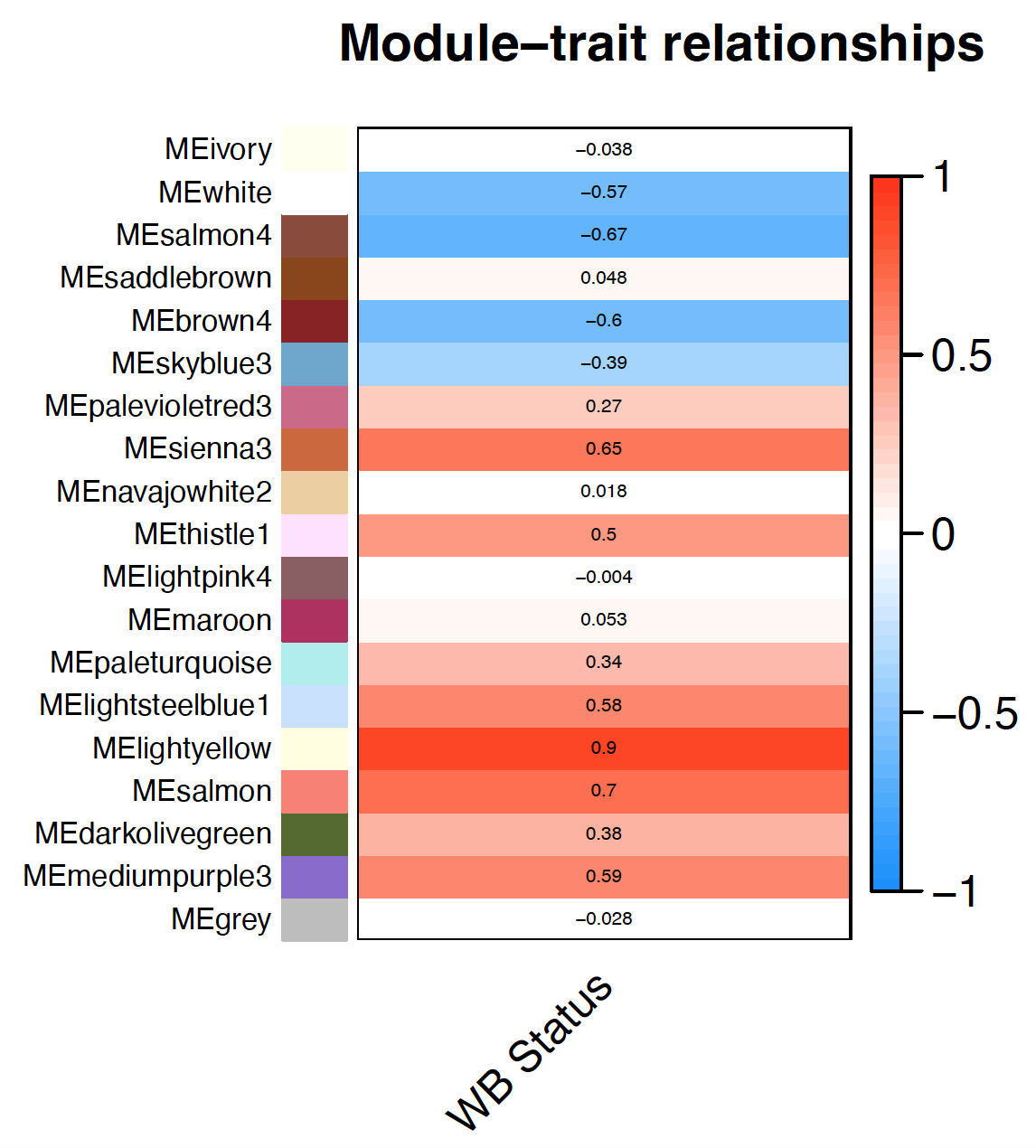
*

*Figure S3: Correlation between modules from weighted gene co-expression network analysis and Wooden Breast (WB) status in broiler chicken pectoralis major muscle. This figure was generated in R v3.5.2 (*[*https://www.R-project.org/*](https://www.R-project.org/)*) using package WGCNA v1.70-3 (*[*http://horvath.genetics.ucla.edu/html/CoexpressionNetwork/Rpackages/WGCNA/*](http://horvath.genetics.ucla.edu/html/CoexpressionNetwork/Rpackages/WGCNA/)*).*

*
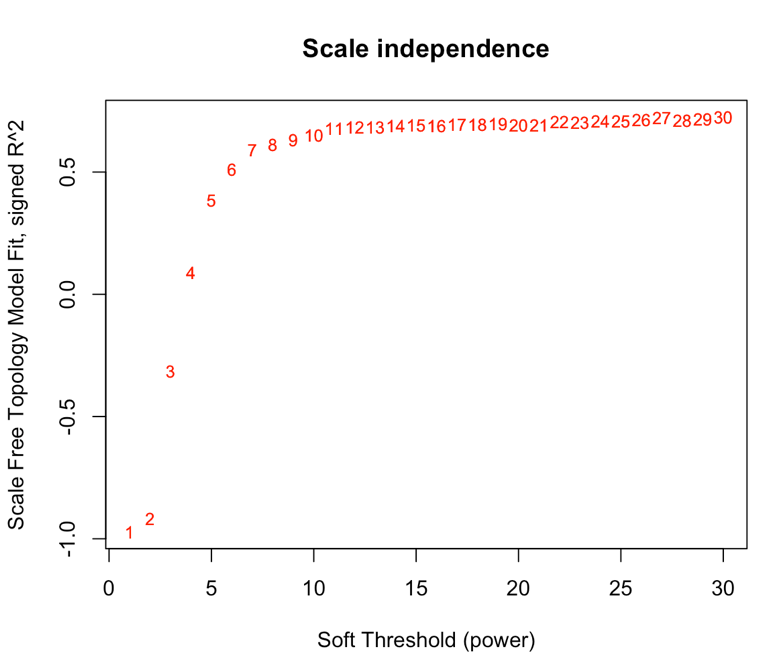

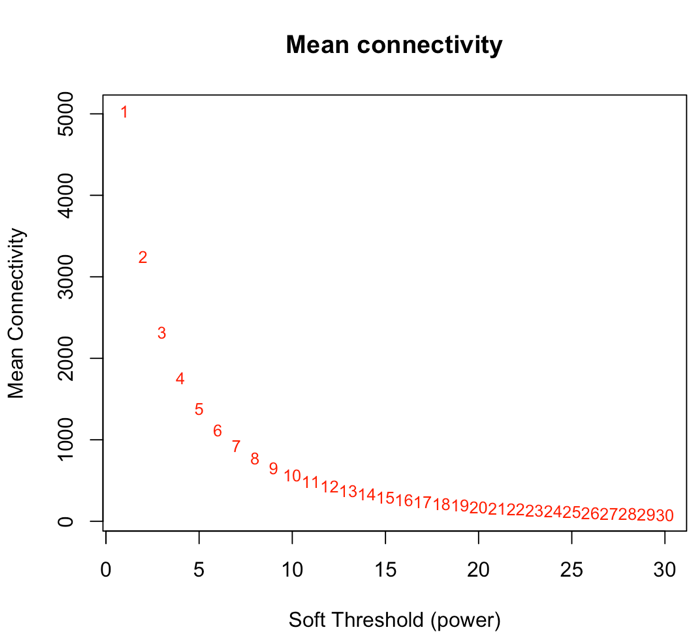
*

*Figure S4: Scale independence (left) and connectivity (right) plot from weighted gene co-expression network analysis in Wooden-Breast-affected and unaffected broiler chickens*

**Table S1. Differentially expressed genes in glycolysis and mitochondrial biosynthesis Wooden Breast (WB) affected breast muscle**

| Ensembl ID | Gene Name | Pathway | Log2FC |
| --- | --- | --- | --- |
| ENSGALG00000013069 | Bisphosphoglycerate Mutase (BPGM) | Glycolysis | ↓1.4 |
| ENSGALG00000034855 | Hexokinase 2 (HK2) | Glycolysis | ↓0.8 |
| ENSGALG00000014442 | Glyceraldehyde-3-Phosphate Dehydrogenase (GAPDH) | Glycolysis | ↓1.6 |
| ENSGALG00000004956 | Glucose-6-Phosphate Isomerase (GPI) | Glycolysis | ↓1.3 |
| ENSGALG00000006300 | Lactate Dehydrogenase A (LDHA) | Glycolysis | ↓1.2 |
| ENSGALG00000014528 | Enolase 2 (ENO2) | Glycolysis | ↑1.4 |
| ENSGALG00000001992 | Pyruvate Kinase L/R (PKLR) | Glycolysis | ↓1.7 |
| ENSGALG00000033271 | Phosphofructokinase, Muscle (PFKM) | Glycolysis | ↓1.5 |
| ENSGALG00000014526 | Triosephosphate Isomerase 1 (TPI1) | Glycolysis | ↓1.7 |
| ENSGALG00000040060 | Nuclear Respiratory Factor 1 (NRF1) | Mitochondrial Biosynthesis | ↓1.1 |

**Table S2. Differentially expressed genes in ECM-receptor interaction in Wooden Breast (WB) affected breast muscle**

| Ensembl ID | Gene Symbol | Gene Name | Log2FC |
| --- | --- | --- | --- |
| ENSGALG00000041555 | COL1A1 | Collagen Type I Alpha 1 Chain | ↑1.8 |
| ENSGALG00000009641 | COL1A2 | Collagen Type I Alpha 2 Chain | ↑1.6 |
| ENSGALG00000005974 | COL6A1 | Collagen Type VI Alpha 1 Chain | ↑1.2 |
| ENSGALG00000039216 | COL6A2 | Collagen Type VI Alpha 2 Chain | ↑1.1 |
| ENSGALG00000003923 | COL6A3 | Collagen Type VI Alpha 3 Chain | ↑1 |
| ENSGALG00000009626 | THBS1 | Thrombospondin 1 | ↑1.5 |
| ENSGALG00000011200 | THBS2 | Thrombospondin 2 | ↑4.1 |
| ENSGALG00000014804 | THBS4 | Thrombospondin 4 | ↑0.7 |
| ENSGALG00000003283 | COMP | Cartilage Oligomeric Matrix Protein | ↑2.1 |
| ENSGALG00000003578 | FN1 | Fibronectin 1 | ↑1.5 |
| ENSGALG00000010926 | SPP1 | Secreted Phosphoprotein 1 | ↑2 |
| ENSGALG00000003589 | VTN | Vitronectin | ↓1.3 |
| ENSGALG00000039990 | TNC | Tenascin C | ↑2 |
| ENSGALG00000004538 | TNN | Tenascin N | ↑1.7 |
| ENSGALG00000010316 | FRAS1 | Fraser Extracellular Matrix Complex Subunit 1 | ↑0.7 |
| ENSGALG00000005426 | FREM1 | FRAS1 Related Extracellular Matrix 1 | ↑1 |
| ENSGALG00000033671 | FREM2 | FRAS1 Related Extracellular Matrix 2 | ↓1.4 |
| ENSGALG00000017272 | VWF | Von Willebrand Factor | ↓0.7 |
| ENSGALG00000054766 | ITGA2B | Integrin alpha-IIb isoform X1 | ↓1.1 |
| ENSGALG00000041577 | ITGA4 | Integrin alpha-4 | ↑0.7 |
| ENSGALG00000034007 | ITGA6 | Integrin alpha-6 | ↑0.9 |
| ENSGALG00000035879 | ITGA7 | Integrin alpha-7 isoform X1 | ↓0.8 |
| ENSGALG00000008747 | ITGA8 | Integrin alpha-8 precursor | ↑2.2 |
| ENSGALG00000002655 | ITGAV | Integrin alpha-V precursor | ↑0.8 |
| ENSGALG00000008007 | ITGA11 | Integrin alpha-11 isoform X2 | ↑1 |
| ENSGALG00000029766 | ITGB5 | Integrin beta-5 precursor | ↑0.6 |
| ENSGALG00000010875 | ITGB8 | Integrin beta-8 isoform X3 | ↑1.8 |
| ENSGALG00000039080 | CD44 | CD44 antigen precursor | ↑0.7 |
| ENSGALG00000008439 | CD36 | CD36 molecule | ↓0.7 |
| ENSGALG00000016480 | SDC1 | Syndecan-1 | ↑0.9 |
| ENSGALG00000015355 | CD47 | leukocyte surface antigen CD47 precursor | ↑0.7 |

**Table S3. Differentially expressed genes in cytokine-cytokine receptor interaction in Wooden Breast (WB) affected breast muscle**

| Ensembl ID | Gene Symbol | Gene Name | Log2FC |
| --- | --- | --- | --- |
| ENSGALG00000002329 | CCL1 | C-C motif chemokine ligand 1 precursor | ↓1.3 |
| ENSGALG00000034478 | CCL4 | C-C motif chemokine 4 homolog precursor | ↓1.3 |
| ENSGALG00000011668 | IL8L1 | Interleukin-8 precursor | ↑1.7 |
| ENSGALG00000006346 | CXCL14 | C-X-C motif chemokine 14 precursor | ↑1.4 |
| ENSGALG00000009603 | PRLL | Prolactin-like protein precursor | ↑6.8 |
| ENSGALG00000007874 | IL18 | Interleukin-18 | ↑1.4 |
| ENSGALG00000009179 | TNFSF10 | Tumor necrosis factor ligand superfamily member 10 | ↑1.4 |
| ENSGALG00000026163 | TNFSF11 | Tumor necrosis factor ligand superfamily member 11 | ↑1.4 |
| ENSGALG00000016852 | TNFSF13B | Tumor necrosis factor ligand superfamily member 13B | ↑1 |
| ENSGALG00000009612 | TGFB2 | Transforming growth factor beta-2 precursor | ↑1.5 |
| ENSGALG00000010346 | TGFB3 | Transforming growth factor beta-3 proprotein preproprotein | ↑2 |
| ENSGALG00000003161 | GDF3 | Embryonic growth/differentiation factor 1 | ↓0.8 |
| ENSGALG00000034616 | INHBA | Inhibin beta A chain precursor | ↑1 |
| ENSGALG00000012787 | BMP6 | Bone morphogenetic protein 6 | ↓0.7 |
| ENSGALG00000007668 | BMP7 | Bone morphogenetic protein 7 | ↑1.2 |
| ENSGALG00000050893 | CCR2 | C-C chemokine receptor type 2 | ↑0.7 |
| ENSGALG00000012357 | CXCR4 | C-X-C chemokine receptor type 4 | ↓1.2 |
| ENSGALG00000042471 | IL2RA | Interleukin-15 receptor subunit alpha precursor | ↑1.3 |
| ENSGALG00000012472 | IL2RB | Interleukin-2 receptor subunit beta precursor | ↓0.7 |
| ENSGALG00000006394 | IL9R | Interleukin-9 receptor isoform X1 | ↓1.3 |
| ENSGALG00000016693 | CSF2RA | Granulocyte-macrophage colony-stimulating factor receptor subunit alpha-like precursor | ↑0.8 |
| ENSGALG00000046156 | LOC101747500 | Granulocyte-macrophage colony-stimulating factor receptor subunit alpha | ↑1.1 |
| ENSGALG00000020316 | IL13RA2 | Interleukin-13 receptor subunit alpha-2 precursor | ↑1.3 |
| ENSGALG00000014855 | GHR | Growth hormone receptor precursor | ↓1 |
| ENSGALG00000003446 | PRLR | prolactin receptor precursor | ↑1 |
| ENSGALG00000028140 | CNTFR | Ciliary neurotrophic factor receptor subunit alpha precursor | ↓1.4 |
| ENSGALG00000030363 | IFNAR1 | Interferon alpha/beta receptor 1 precursor | ↑0.6 |
| ENSGALG00000014477 | CD4 | T-cell surface glycoprotein CD4 precursor | ↑0.8 |
| ENSGALG00000011442 | TGFBR2 | TGF-beta receptor type-2 precursor | ↑0.9 |
| ENSGALG00000041257 | ACVR1C | Activin receptor type-1C isoform X4 | ↓1.5 |

**Table S4. Differentially expressed genes in Wnt signaling pathways in Wooden Breast (WB) affected breast muscle**

| Ensembl ID | Gene Symbol | Gene Name | Log2FC |
| --- | --- | --- | --- |
| ENSGALG00000043009 | WNT1 | Protein Wnt-1 | ↑1.3 |
| ENSGALG00000000839 | WNT11 | Protein Wnt-11 precursor | ↑1.3 |
| ENSGALG00000012998 | WNT5B | Protein Wnt-5B precursor | ↓1 |
| ENSGALG00000003015 | SERPINF1 | Pigment epithelium-derived factor precursor | ↑0.9 |
| ENSGALG00000009241 | SFRP2 | Secreted frizzled-related protein 2 precursor | ↑1.9 |
| ENSGALG00000031997 | SFRP4 | Secreted frizzled-related protein 4 isoform X1 | ↑1.8 |
| ENSGALG00000002763 | FRZB | Secreted frizzled-related protein 3 precursor | ↑0.7 |
| ENSGALG00000042607 | RSPO3 | R-spondin-3 precursor | ↑0.9 |
| ENSGALG00000009064 | FZD1 | Frizzled-1 precursor | ↑1.4 |
| ENSGALG00000041927 | FZD9 | Frizzled-9 precursor | ↓0.7 |
| ENSGALG00000007543 | APCDD1L | Protein APCDD1-like | ↑1.3 |
| ENSGALG00000001600 | DVL1 | Segment polarity protein dishevelled homolog DVL-1 isoform X1 | ↓0.7 |
| ENSGALG00000003767 | NKD1 | Protein naked cuticle homolog 1 isoform X2 | ↑1.1 |
| ENSGALG00000039200 | AXIN2 | Axin-2 | ↑0.7 |
| ENSGALG00000000220 | APC | Adenomatous polyposis coli protein isoform X3 | ↑0.6 |
| ENSGALG00000006480 | TCF7 | Transcription factor 7 | ↑1.3 |
| ENSGALG00000031960 | LEF1 | Lymphoid enhancer-binding factor 1 | ↑2 |
| ENSGALG00000007762 | CREBBP | CREB-binding protein isoform X1 | ↓0.6 |
| ENSGALG00000017283 | CCND2 | G1/S-specific cyclin-D2 | ↑0.9 |
| ENSGALG00000003485 | CCND3 | G1/S-specific cyclin-D3 | ↓0.7 |
| ENSGALG00000030115 | WISP1 | WNT1-inducible-signaling pathway protein 1 precursor | ↑1.7 |
| ENSGALG00000003916 | SIAH1 | E3 ubiquitin-protein ligase SIAH1 | ↓1.3 |
| ENSGALG00000002229 | FBXW11 | F-box/WD repeat-containing protein 11 | ↓0.6 |
| ENSGALG00000011993 | RBX1 | E3 ubiquitin-protein ligase RBX1 | ↑0.7 |
| ENSGALG00000011019 | ROR1 | Inactive tyrosine-protein kinase transmembrane receptor ROR1 | ↓0.5 |
| ENSGALG00000009556 | PRICKLE1 | Prickle-like protein 1 | ↑0.7 |
| ENSGALG00000007332 | PRICKLE2 | Prickle-like protein 2 isoform X2 | ↑0.8 |
| ENSGALG00000012017 | DAAM1 | T-cell surface glycoprotein CD4 precursor | ↓0.7 |
| ENSGALG00000016451 | ROCK2 | Rho-associated protein kinase 2 isoform X1 | ↓0.7 |
| ENSGALG00000005621 | CAMK2A | Calcium/calmodulin-dependent protein kinase type II subunit alpha | ↑0.5 |
| ENSGALG00000041635 | CAMK2D | Calcium/calmodulin-dependent protein kinase type II delta chain | ↓0.5 |

**Table S5. Differentially abundant metabolites in Wooden Breast (WB) affected and unaffected breast muscle**

| Metabolite | FDR | Average Affected* | Average Unaffected* |
| --- | --- | --- | --- |
| Threonine | 2.19E-07 | 0.86 | -1 |
| 4-hydroxybutyrate (GHB) | 1.52E-05 | 0.62 | -1.4 |
| Taurine | 1.53E-05 | 0.84 | -1 |
| Glycine | 1.79E-05 | 0.82 | -0.98 |
| N-acetylmethionine | 3.42E-05 | 0.83 | -1 |
| Sarcosine (N-Methylglycine) | 4.43E-05 | 0.8 | -0.96 |
| UDP-glucuronate | 4.47E-05 | 0.6 | -1.34 |
| UDP-acetylglucosamine | 4.59E-05 | 0.81 | -0.97 |
| 5-oxoproline | 5.88E-05 | 0.82 | -0.98 |
| Anserine | 5.93E-05 | -0.83 | 1 |
| Glycerophosphorylcholine (GPC) | 6.57E-05 | 0.8 | -0.96 |
| Sedoheptulose-7-phosphate | 7.32E-05 | 0.8 | -0.96 |
| Guanosine 5'- monophosphate (5'-GMP) | 9.88E-05 | 0.79 | -0.95 |
| Glycerol 3-phosphate (G3P) | 0.00012 | 0.79 | -0.95 |
| Palmitoleate (16:1n7) | 0.0001473 | 0.78 | -0.94 |
| Pyroglutamine | 0.0001477 | -0.82 | 0.98 |
| Carnitine | 0.00015 | -0.79 | 0.94 |
| Guanosine | 0.00017 | 0.80 | -0.96 |
| Histidine | 0.000175 | 0.78 | -0.93 |
| Phosphoethanolamine | 0.00026 | 0.78 | -0.94 |
| N-acetylaspartate (NAA) | 0.00028 | 0.79 | -0.94 |
| Docosadienoate (22:2n6) | 0.00031 | 0.78 | -0.94 |
| Asparagylisoleucine | 0.00032 | -0.99 | 0.85 |
| Serine | 0.00035 | 0.76 | -0.91 |
| Flavin adenine dinucleotide (FAD) | 0.00035 | 0.79 | -0.95 |
| Histamine | 0.00038 | 0.78 | -0.93 |
| Ribulose | 0.0004 | 0.31 | -1.85 |
| Isobutyrylcarnitine | 0.00041 | -0.79 | 0.94 |
| Malate | 0.00046 | 0.78 | -0.94 |
| 2-palmitoylglycerophosphocholine | 0.00046 | 0.76 | -0.91 |
| Carnosine | 0.00048 | -0.78 | 0.93 |
| Serylisoleucine | 0.00062 | -1.3 | 0.59 |
| Glycerophosphoethanolamine | 0.00065 | 0.75 | -0.9 |
| Uridine monophosphate (5' or 3') | 0.00072 | 0.75 | -0.9 |
| C-glycosyltryptophan | 0.00073 | 0.75 | -0.89 |
| Chiro-inositol | 0.00074 | 0.75 | -0.9 |
| Cytidine | 0.00086 | 0.75 | -0.9 |
| Glutamine | 0.00097 | 0.74 | -0.88 |
| N6-acetyllysine | 0.00099 | 0.39 | -1.65 |
| Isovalerylcarnitine | 0.001054 | -0.74 | 0.88 |
| 15-HETE | 0.001056 | 0.74 | -0.89 |
| Asparagine | 0.00114 | -0.77 | 0.92 |
| 13-HODE + 9-HODE | 0.00116 | 0.75 | -0.9 |
| Adenine | 0.00118 | 0.74 | -0.89 |
| N-acetylcarnosine | 0.0012 | -0.73 | 0.87 |
| Xylitol | 0.00127 | 0.63 | -1.1 |
| 6-phosphogluconate | 0.001293 | 0.74 | -0.89 |
| Lactate | 0.001294 | -0.77 | 0.92 |
| Pyruvate | 0.0013 | -0.75 | 0.9 |
| Fumarate | 0.00133 | 0.77 | -0.92 |
| Inosine 5'-monophosphate (IMP) | 0.00135 | -0.77 | 0.92 |
| 2-palmitoylglycerol (2-monopalmitin) | 0.00136 | -0.9 | 0.79 |
| 5-methylthioadenosine (MTA) | 0.00137 | 0.74 | -0.89 |
| Table S5 (continued). Differentially abundant metabolites in Wooden Breast (WB) affected and unaffected breast muscle | | | |
| Metabolite | **FDR** | **Average Affected*** | **Average Unaffected*** |
| 1-methylhistidine | 0.0014 | 0.73 | -0.88 |
| Adenosine 2'-monophosphate (2'-AMP) | 0.0015 | 0.55 | -1.2 |
| Myo-inositol | 0.0016 | 0.73 | -0.88 |
| Cystathionine | 0.00169 | 0.72 | -0.86 |
| Cytidine-3'-monophosphate (3'-CMP) | 0.0018 | -0.73 | 0.87 |
| Stearoyl sphingomyelin | 0.002 | -0.72 | 0.86 |
| Eicosenoate (20:1n9 or 11) | 0.0021 | 0.75 | -0.9 |
| Proline | 0.0022 | -0.71 | 0.85 |
| 3-methylhistidine | 0.0023 | 0.36 | -1.4 |
| 1-linoleoylglycerol (1-monolinolein) | 0.00241 | 0.71 | -0.85 |
| Methionine | 0.00242 | 0.7 | -0.85 |
| Alanyltyrosine | 0.0026 | -1.1 | 0.64 |
| Uracil | 0.00279 | 0.7 | -0.84 |
| 15-KETE | 0.0028 | 0.72 | -0.87 |
| Ectoine | 0.0029 | -0.73 | 0.87 |
| Palmitate (16:0) | 0.003 | 0.72 | -0.87 |
| Myristate (14:0) | 0.0031 | 0.69 | -0.83 |
| Serylphenyalanine | 0.00329 | -0.69 | 0.83 |
| Adenosine 5'diphosphoribose | 0.0033 | -0.76 | 0.92 |
| Xanthine | 0.0036 | 0.69 | -0.83 |
| N-acetylthreonine | 0.0036 | 0.69 | -0.83 |
| Adenosine 5'-monophosphate (AMP) | 0.0039 | -0.69 | 0.83 |
| Cysteine-glutathione disulfide | 0.004 | 0.68 | -0.82 |
| Pro-hydroxy-pro | 0.0041 | 0.75 | -0.9 |
| Serylvaline | 0.0042 | -1.25 | 0.5 |
| Creatinine | 0.0045 | -0.7 | 0.84 |
| Hexadecanedioate | 0.005 | 0.68 | -0.81 |
| Hypoxanthine | 0.0051 | 0.71 | -0.86 |
| Uridine | 0.0052 | 0.71 | -0.85 |
| Glutathione, reduced (GSH) | 0.0057 | 0.72 | -0.86 |
| Tyrosine | 0.006 | 0.68 | -0.82 |
| Inositol 1-phosphate (I1P) | 0.0061 | 0.66 | -0.8 |
| Leucine | 0.0066 | 0.66 | -0.79 |
| Aspartate | 0.0074 | 0.67 | -0.8 |
| 2-linoleoylglycerol (2-monolinolein) | 0.0083 | 0.65 | -0.78 |
| Cholesterol | 0.0084 | 0.67 | -0.81 |
| Glycerol 2-phosphate | 0.0088 | 0.64 | -0.77 |
| Phosphate | 0.0089 | -0.66 | 0.79 |
| Urate | 0.00936 | 0.67 | -0.80 |
| Valine | 0.0094 | 0.64 | -0.76 |
| Dihomo-linolenate (20:3n3 or n6) | 0.0099 | 0.64 | -0.77 |
| Hexanoylcarnitine | 0.01 | -0.64 | 0.77 |
| 3-hydroxybutyrate (BHBA) | 0.0103 | 0.63 | -0.75 |
| Glutamate | 0.01032 | 0.63 | -0.76 |
| 10-heptadecenoate (17:1n7) | 0.01061 | 0.64 | -0.77 |
| Alanine | 0.01064 | 0.63 | -0.75 |
| Cytidine 5'-monophosphate (5'-CMP) | 0.0109 | 0.71 | -0.85 |
| 2-hydroxybutyrate (AHB) | 0.0113 | 0.56 | -1.05 |
| 3-(4-hydroxyphenyl)lactate | 0.0114 | 0.62 | -0.75 |
| 1-stearoylglycerophosphocholine (18:0) | 0.012 | 0.64 | -0.76 |
| Thymidine | 0.013 | 0.64 | -0.76 |
| Ascorbate (Vitamin C) | 0.014 | 0.64 | -0.77 |
| Table S5 (continued). Differentially abundant metabolites in Wooden Breast (WB) affected and unaffected breast muscle | | | |
| Metabolite | **FDR** | **Average Affected*** | **Average Unaffected*** |
| 2-hydroxystearate | 0.0146 | 0.61 | -0.73 |
| 2-docosapentaenoylglycerophosphoethanolamine | 0.015 | 0.61 | -0.73 |
| 2-palmitoylglycerophosphoethanolamine | 0.016 | 0.66 | -0.79 |
| Arginine | 0.017 | 0.62 | -0.74 |
| 1-linolenoylglycerol | 0.0171 | 0.6 | -0.72 |
| Isoleucine | 0.0175 | 0.6 | -0.72 |
| 1-palmitoylglycerol (1-monopalmitin) | 0.0176 | 0.63 | -0.75 |
| Phenylalanine | 0.0178 | 0.6 | -0.72 |
| Glycerate | 0.018 | -0.84 | 0.62 |
| 3-aminoisobutyrate | 0.0184 | 0.62 | -0.74 |
| Docosapentaenoate (n6 DPA; 22:5n6) | 0.019 | -0.6 | 0.71 |
| Fructose 1,6-diphosphate | 0.022 | -0.79 | 0.64 |
| 2-hydroxypalmitate | 0.024 | 0.62 | -0.74 |
| 1-(5'-Phosphoribosyl)-5-amino-4-imidazolecarboxamide | 0.036 | 0.59 | -0.71 |

*Average standardized value of the metabolite
